# Improvement of Impulsivity and Decision Making by Transcranial Direct Current Stimulation of the Dorsolateral Prefrontal Cortex in a Patient with Gambling Disorder

**DOI:** 10.1007/s10899-021-10050-1

**Published:** 2021-07-02

**Authors:** Adriana Salatino, Roberta Miccolis, Roberto Gammeri, Marco Ninghetto, Francesco Belli, Marcello Nobili, André Mouraux, Raffaella Ricci

**Affiliations:** 1 SAMBA - SpAtial, Motor and Bodily Awareness Research Group, Department of Psychology, University of Turin, Via Po 14, 10123 Turin, Italy; 2 Division Cognition and Systems (COSY), Institute Institute of Neuroscience (IONS), Université Catholique de Louvain (UCL), Brussels, Belgium; 3 Neuroplasticity Laboratory, Nencki Institute for Experimental Biology, Polish Academy of Sciences, Warsaw, Poland; 4 Potsdam Embodied Cognition Group (PECoG), Psychology Department, University Potsdam, Karl-Liebknecht-Strasse 24-25, House 14, 14476 Potsdam, Germany; 5 Academy of Medicine of Turin, Turin, Italy; 6 NIT - Neuroscience Institute of Turin, Via Verdi, 8, 10124 Turin, Italy

**Keywords:** Gambling disorder, tDCS, DLPFC, Decision making, Impulsivity

## Abstract

Gambling disorder (GD) is a form of behavioral addiction. In recent years, it has been suggested that the application of transcranial Direct Current Stimulation (tDCS) to the dorsolateral prefrontal cortex (DLPFC), which plays a key role in top-down inhibitory control and impulsivity, may represent a new therapeutic approach for treating addictions. Here we investigated the effectiveness of a novel low dose tDCS protocol (i.e. six sessions of right anodal/left cathodal tDCS for 20 min, with a current intensity of 1 mA) applied to DLPFC in a patient with GD. To evaluate the effect of the proposed intervention, cognitive, psychological and behavioural evaluations were performed at different time points, pre and post intervention. The results showed improvement of impulsivity, decision making, and cognitive functioning after tDCS intervention. Findings of the present study suggest that low doses of right anodal/left cathodal tDCS to DLPFC may effectively improve gambling behaviour. They also suggest to carefully evaluate the effects of this tDCS polarity on the patient’s emotional state. The current protocol warrants further investigation in large groups of patients, as it may provide relevant insights into the design of effective, low dose treatments of gambling disorder.

## Background

Gambling disorder (GD) is a form of behavioral addiction, recently assigned to the category of ‘substance-related and addictive disorders’ in the DSM-5 (American Psychiatric Association, 2013). Both GD and addictive disorders show mesolimbic abnormal dopaminergic activity in association with impulsivity, craving, and impaired decision making (Feil & Zangen, 2010). Non-invasive brain stimulation (NIBS) techniques have been proposed as potential treatment for several neurological and neuropsychiatric disorders (George, 2005; George et al., 2014; Salatino et al., 2014a and b; D’Agata et al., 2016; Salatino et al., 2019). Novel therapeutic NIBS approaches have been also investigated with the aim of reducing craving and impulsivity in addiction (Grall-Bronnec & Sauvaget, 2014; Jansen et al., 2013). Some of them apply transcranial Direct Current Stimulation (tDCS) to the dorsolateral prefrontal cortex (DLPFC), a site that plays a key role in top-down inhibitory control and reward (Lefaucheur et al., 2017). For example, Soyata and colleagues, (2019), administering three sessions (on alternate days) of left cathodal/right anodal tDCS (20 min, 2 mA) or sham stimulation to bilateral DLPFC in GD patients found, after the last session of active tDCS, improved decision making and cognitive flexibility, as measured by the Iowa Gambling test (IGT) and the Wisconsin Card Sorting Test (WCST), respectively. Recently, Martinotti and colleagues (2018) stimulated left and right DLPFC to control craving and impulsivity, in a 26 years old male with GD and substance-use disorders (SUDs). In this study the authors applied ten twice-daily sessions of stimulation (20 min, 1.5 mA). Each day, left anodal/right cathodal followed by right anodal/left cathodal tDCS was applied to bilateral DLPFC. The two types of protocol were expected to be beneficial in controlling craving (left anodal DLPFC stimulation) and emotional impulses (right anodal DLPFC stimulation). As expected, the ten days tDCS treatment improved the patient’s psychiatric symptomatology and the severity of gambling and craving symptoms.

Based on the above preliminary evidence, we explored the effectiveness of a low dose tDCS treatment in a GD patient using a novel protocol. We assessed tDCS effects on impulsivity and decision making using different psychological and cognitive tests. We also administered, for the first time, a Go/No-go task, to evaluate changes in response inhibition.

## Case Report

The patient is a 45 years old right-handed man (21 years of education) with a 5-year history of Gambling Disorder (GD). Pharmacological treatment (alprazolam 1.5 mg/d, trazodone 0.65 mg/d) was maintained constant throughout the study. He reports that during the last five years gamble was the only way to calm anxiety.

The patient underwent a cognitive and psychological assessment at four time points: one week before (T0) and just before the treatment (T1), the day after the last application (T2), and after two weeks from the end of the treatment (T3). He was assessed against inclusion/exclusion criteria for the use of tDCS and signed a written informed consent. He was informed about the general purpose of the treatment. The treatment consisted of six left cathodal/right anodal tDCS applications (once a day, on alternate days, for two weeks) to the DLPFC for 20 min with a current intensity of 1 mA. TDCS was delivered by a battery driven constant current stimulator (HDC stim, HDC kit, Magstim Company Limited, Whitland, Wales, UK) using a pair of surface saline-soaked sponge electrodes (5 × 5 cm). During the last 3 min of each session, the IOWA gambling task was administered to assess tDCS effects on patient’s decision-making, but also to potentiate its effect, in line with previous evidence (Martin et al., 2014, but see also Dedoncker et al., 2016) showing that ‘online’ tDCS (i.e. during performance) may enhance cognitive skills acquisition. The patients' anxiety levels, impulsivity in everyday life and quality of life were evaluated using the Hamilton Rating Scale for Depression (HAM-D), the Barratt Impulsiveness scale (BIS-11) and the Short Form Test–36 (SF-36), administered at T0 and T3. His global cognitive functioning was also assessed at T0 and T3 using the Montreal Cognitive Assessment (MoCA). In addition, at T0 and T3 specific gambling tests were administered: the South Oaks Gambling Screen (SOGS) (Bonini et al., 2018) and the Canadian Problem Gambling Index (CPGI) (Bonnaire& Barrault, 2018). During T1, T2 and T3, the patient completed three computerized tasks, measuring decision making and impulsivity: the Balloon Analogue Risk task (Bonini et al., 2018), the IOWA Gambling task (Li et al., 2010; Bechara et al., 1997), and a Go/No-go task (Bezdjian et al., 2009). Finally, since altered activity of dorsal prefrontal cortex may also affect spatial attention (Ninghetto et al., 2019; Ricci et al., 2012, 2014), at each time point, the patient also performed a line bisection task (ten 20 cm long horizontal lines).

## Results

Results are reported in Table 1. They show a reduction of gambling scores on the SOGS (from 10 at T0 to 6 at T3) and the CPGI (from 22 at T0 to 11 at T3) and of impulsivity on the motor factor of the BIS-11 (from 33 at T0 to 21 at T3) from T0 to T3. Cognitive functioning, as measured by the MoCA, was also found to be improved from T0 (23) to T3 (29), especially in relation to the memory domain. At T3, the patient also showed amelioration in 2 of the 9 SF-36 subscales (i.e. role-physical and physical functioning), but worsening in 3 of the 9 SF-36 subscales (i.e. role-emotional, body pain, and social functioning). The HAM-D also indicated a light worsening of anxiety at 2 weeks from treatment (T3). For the computer tasks significant differences were found between T1, T2 and T3. In particular, for the BART the repeated measures ANOVA [F_2, 110_ = 9.713; *p* = 0.0001] revealed a significant effect of session. Duncan post-hoc analyses revealed significant differences between T1 and T2 (*p* = 0.0002) and between T2 and T3 (*p* = 0.0005). Specifically, performance improved (i.e. the number of unexploded balloons pumps decreased) after treatment but returned to baseline level at follow-up. The patient also showed a significant improvement on the Go/No-go task, as indexed by faster RTs (Wilcoxon test with Bonferroni correction, *p* = 0.003) in T2 compared to T1 (with no changes in omissions/commissions). No significant differences between sessions were observed on the IOWA Gambling test.

**Table 1:** Cognitive and emotive evaluations before treatment (T0 and T1), after one day (T2, i.e. after 13 days from T1) and two weeks (T3) from the treatment end

		T0	T1	T2	T3
	Cut-off				
MoCA	< 26: cognitive impairment	23/30	–	–	29/30
SF36 (0 100%) *PF*		70	–	–	85
*RF*		50	–	–	100
*BP*		56.67	–	–	34.44
*GH*		50	–	–	50
*VT*		50	–	–	50
*SF*		50	–	–	37.5
*RE*		33.33	–	–	0
*MH*		56	–	–	56
*CH*		75	–	–	75
HAM-A	_ < 17: mild anxiety severity _ 18–24: mild to moderate anxiety severity _ 25–30: moderate to severe anxiety severity	37	–	–	41
Line Bisection Task		−0.4 cm	−0.4 cm	−0.33 cm	−0.4 cm
SOGS	5 or more: Probable pathological gambler	10	–	–	6
The CPGI	_3-7: problematic gambler _8 or more: pathological gambler	22	–	–	11
BIS-11 *Attentive*	_ minimum: 8 _ maximum: 32	23	–	–	23
*Motor*	_ minimum: 11 _maximum: 44	33	–	–	21
*Non planning*	_minimum: 11 _ maximum: 44	35	–	–	34
*Total score*	_ minimum: 30 _maximum: 120	91	–	–	78
BART *(average of unexploded balloons’ pumps)*			Mean: 4.77 SD: 3.05	Mean: 3.07 SD: 1.86	Mean: 4.66 SD: 2.77
GO-NOGO *(RT)*			Mean: 526.42 SD: 233.78	Mean: 440.79 SD: 67.35	Mean: 492.72 SD: 154.26
IGT (*n° trials*)					
(A + B)			44	43	43
(C + D)			56	57	57

SF-36 Short Form Health Survey (0  100%): scores expressed as percentage, with a higher score indicating a better health related quality of life; Physical Functioning (PF), Role-Physical (RF), Bodily Pain (BP), General Health (GH), Vitality (VT), Social Functioning (SF), Role-Emotional (RE), Mental Health (MH), Changing in Health state (CH); HAM-A: Hamilton Anxiety Rating Scale; SOGS: South Oaks Gambling Screen; CPGI: Canadian Problem Gambling Index; BIS-11: Barratt Impulsiveness Scale; BART: Balloon Analogue Risk Taking task; IGT: Iowa Gambling Task

## Conclusion

Findings of the present study show that the proposed tDCS treatment improved patient’s pathological gambling propensity as evaluated by self-assessment measures (SOGS and CPGI), likely via reduction of impulsivity, as revealed by both objective and subjective assessments, and improved decision-making deficits, as measured by behavioral tasks. Specifically, the patient showed reduced impulsivity on BIS-11 at two weeks from the end of treatment (at T3) and improved ability to inhibit a response on the Go/No-go task soon after treatment (at T2, 13 days from T1, i.e. before the beginning of the treatment). However, this improvement was not maintained at follow-up (i.e. 14 days from the end of treatment). A similar result was observed for decision making that improved on the BART task soon after treatment (T2) but returned to baseline level at follow-up (T3). These findings suggest that the patient’s improvements observed at T2 were very likely due to the effect of the treatment, and not to other factors such as, for example, a learning effect. Surprisingly, tDCS did not affect decision making on the IOWA Gambling test. Differences in the outcomes of the BART and the IOWA might be due to differences in the brain regions mainly involved by the two tasks. There is evidence that BART performance mainly engages DLPFC (Rao et al., 2008)—that, in our study, was targeted by tDCS—while IOWA performance primarily engages basal ganglia, orbitofrontal and ventromedial prefrontal cortices (Lawrence et al., 2009; Li et al., 2010; Power et al., 2012). Alternatively, the different outcomes might be explained by differences in task sensitivity. Despite at follow-up the patient showed amelioration in quality of life subscales (SF-36) concerning physical functioning, he scored worse on the subscales related to emotional, bodily and social functioning. This finding, together with the observation of increased anxiety (HAM-A) at follow-up, seem to suggest a negative effect of this treatment on the patient’s mood and emotional state. This possibility is in line with the evidence that the application of the opposite (left anodal/right cathodal) polarity to DLPFC is effective in treating depression and mood disorders (Ninghetto et al., 2019). Finally, this tDCS protocol did not affect patient’s spatial attention consistent with previous evidence showing attention modulation by an opposite tDCS polarity (see for example Ninghetto et al., 2019, but also Sarasso et al, 2018). Importantly, the patient’s overall cognitive decline was significantly improved by tDCS intervention.

To summarize, the present study suggests that repeated sessions of left cathodal/right anodal tDCS applied to the DLPFC may improve impulsivity, decision making, and gambling behavior, besides enhancing the overall cognitive functioning. On the other hand, our findings also suggest that this tDCS polarity might not be beneficial for anxiety and emotional aspects.

To the best of our knowledge, this is the first report of tDCS applied for few sessions with low current intensity, leading to symptoms amelioration in GD. Despite protocol differences, our findings, in line with previous evidence (Martinotti et al., 2018; Soyata et al., 2019), show that bilateral stimulation of DLPFC may ameliorate gambling behavior likely through improvement of impulsivity and decision making (Martinotti et al., 2018). They also suggest to carefully evaluate the possible impact of this tDCS polarity on patient’s mood and/or emotional aspects.

This case study has two main limitations: (1) there was no sham control condition, and (2) the patient was not blind to the potential treatment effect. Both limitations make difficult to draw definitive conclusions from the present study. To overcome these limitations, the present findings need to be further investigated in large groups of patients, using double-blind sham-controlled designs. However, despite these limitations, our findings may be relevant for the design of new therapeutic protocols, adopting low stimulation doses for the treatment of GB patients.
